# Mobile Phone-Connected Wearable Motion Sensors to Assess Postoperative Mobilization

**DOI:** 10.2196/mhealth.3785

**Published:** 2015-07-28

**Authors:** Geoff Appelboom, Blake E Taylor, Eliza Bruce, Clare C Bassile, Corinna Malakidis, Annie Yang, Brett Youngerman, Randy D'Amico, Sam Bruce, Olivier Bruyère, Jean-Yves Reginster, Emmanuel PL Dumont, E Sander Connolly Jr

**Affiliations:** ^1^Cerebrovascular LabColumbia University Medical CenterNew York, NYUnited States; ^2^Department of NeurosurgeryColumbia UniversityNew York, NYUnited States; ^3^College of Physicians and SurgeonsColumbia UniversityNew York, NYUnited States; ^4^Department of Rehabilitation and Regenerative MedicineColumbia University Medical CenterNew York, NYUnited States; ^5^Support Unit in Epidemiology and BiostatisticsUniversity of LiègeLiègeBelgium; ^6^Epidemiology and Health EconomicsDepartment of Public HealthUniversity of LiègeLiègeBelgium; ^7^The Joan and Irwin Jacobs TechnionCornell Innovation InstituteCornell UniversityNew York, NYUnited States; ^8^Neurocritical CareColumbia University Medical CenterNew York, NYUnited States

**Keywords:** mobilization, activity tracking, postoperative, physiotherapy, functional recovery, physical therapy, gait, neurorehabilitation

## Abstract

**Background:**

Early mobilization after surgery reduces the incidence of a wide range of complications. Wearable motion sensors measure movements over time and transmit this data wirelessly, which has the potential to monitor patient recovery and encourages patients to engage in their own rehabilitation.

**Objective:**

We sought to determine the ability of off-the-shelf activity sensors to remotely monitor patient postoperative mobility.

**Methods:**

Consecutive subjects were recruited under the Department of Neurosurgery at Columbia University. Patients were enrolled during physical therapy sessions. The total number of steps counted by the two blinded researchers was compared to the steps recorded on four activity sensors positioned at different body locations.

**Results:**

A total of 148 motion data points were generated. The start time, end time, and duration of each walking session were accurately recorded by the devices and were remotely available for the researchers to analyze. The sensor accuracy was significantly greater when placed over the ankles than over the hips (*P*<.001). Our multivariate analysis showed that step length was an independent predictor of sensor accuracy. On linear regression, there was a modest positive correlation between increasing step length and increased ankle sensor accuracy (*r*=.640, *r* ^2^=.397) that reached statistical significance on the multivariate model (*P*=.03). Increased gait speed also correlated with increased ankle sensor accuracy, although less strongly (*r*=.444, *r* ^2^=.197). We did not note an effect of unilateral weakness on the accuracy of left- versus right-sided sensors. Accuracy was also affected by several specific measures of a patient’s level of physical assistance, for which we generated a model to mathematically adjust for systematic underestimation as well as disease severity.

**Conclusions:**

We provide one of the first assessments of the accuracy and utility of widely available and wirelessly connected activity sensors in a postoperative patient population. Our results show that activity sensors are able to provide invaluable information about a patient’s mobility status and can transmit this data wirelessly, although there is a systematic underestimation bias in more debilitated patients.

## Introduction

Functional recovery refers to improvement in mobility and independence of activities of daily living (ADL) after hospitalization for surgery or acute illness. It is a widely used outcome measure, especially in postoperative patients and in those with neurological conditions. Mobilization is a cornerstone of rehabilitation therapy not only in the hospital and acute care settings, but also at home and in the community [1]. Whereas close supervision and monitoring generally allow health care professionals to track improvement in hospitalized patients, objective measures of recovery in the outpatient setting are lacking [2]. Novel and affordable physical activity sensors may finally provide such a measure, but their accuracy in patients with limited mobility is variable and the protocols for using them are not standardized.

Early in-hospital mobilization reduces the risk of conditions related to prolonged bed rest—pulmonary embolism, atelectasis, pneumonia, decubitus ulcers—and is associated with improved survival, decreased length of hospitalization, and improved psychological well-being [3-5]. Not only are many of these benefits seen in postoperative neurosurgical patients—both spine and cranial—but also in patients recovering from joint replacements, cardiac surgery, stroke, breast cancer, and those in the intensive care unit (ICU) [6-11]. Increased mobilization in the outpatient setting is associated with improved survival and functional status, and the degree of mobilization may be quantified by measures such as gait speed, which itself correlates with survival [12-16].

Commercially available activity sensors have tremendous potential to provide this data because recent technological advances have resulted in devices that are small, wearable, affordable, and able to relay their data wirelessly via patient mobile phones or wireless networks at home or in the hospital [15,16]. Certain sensors contain accelerometers, which measure physical activity—number of steps taken, distance ambulated, gait velocity—by calculating body movements in one, two, or three orthogonal planes [17]. They record continuously for days to weeks and produce data that, in turn, may be used to interpret the duration, intensity, frequency, and variations of the patient’s physical activity over time. Most significantly, this data can be collected and analyzed in real time while the patient is in his or her home environment.

However, there is little consensus on how to use activity sensors to provide an *accurate* measure of patient mobility. To address this issue, we sought to evaluate the usability for remote assessment and accuracy of a common, widely used activity sensor to quantify postoperative mobility. We hypothesized that a wearable motion sensor may vary in accuracy depending on where it is positioned on the patient’s body [18] and the patient’s degree of disability as it relates to gait [19,20].

## Methods

### Ethical Approval

This study complies with the Declaration of Helsinki. This research protocol has been approved by the Columbia University Medical Center Institutional Review Board (IRB) (protocol number AAA-M6702).

### Study Population

A total of 27 consecutive subjects were prospectively recruited from a convenience sample of inpatients under the Department of Neurosurgery at Columbia University from November 2013 to July 2014. The patient subjects were a median of 3 days postoperative and were enrolled during their first or second inpatient physical therapy session provided by the Department of Rehabilitation and Regenerative Medicine. Of the 27 patients, 20 (74%) were postoperative spine patients (primarily laminoplasties, laminectomies, and microdiscectomies) and 7 (26%) had had craniotomies for tumors or vascular malformations. Additional patient characteristics are included in Table 1 in the Results section. Inclusion criteria were patients who were ambulatory prior to hospitalization, able to follow commands, and able to ambulate at least 4 meters without stopping during the physical therapy session. Exclusion criteria were patients with extrapyramidal disorders, significant visual impairment, severe and debilitating pain, severe sensory neuropathies, vestibular dysfunction, and patients under 18 years of age. The study was approved by the Columbia University Medical Center IRB, and each subject provided informed consent for participation in the study. Each patient received full neurosurgical standard of care, and patient health information used in the study was used in accordance with Health Insurance Portability and Accountability Act (HIPAA) privacy policies.

A total of 10 healthy volunteer controls with no preexisting gait abnormalities were also included in the study as a comparison. Their characteristics can also be found in Table 1.

### Instrument

The activity sensor used was the FitBit Zip (produced by FitBit, San Francisco, CA). The device records data such as the number of steps taken and the time stamps of when these steps occurred, and automatically syncs to mobile phones (and other devices) via Bluetooth. The recorded data is uploaded online to a user-friendly personalized account, and is easily searchable by date and time with a resolution of 15-minute time intervals. FitBit is considered one of the leaders in the market of wearable activity sensors, and at a cost of under US $60, the Zip model is far more affordable than comparable devices [21]. The device detects movement by using a built-in 3-axis accelerometer and, according to the company, it may be worn in several locations including on a belt, in a pocket, or over the chest using an attachable clip. Time is recorded by a built-in clock, which syncs to the mobile phone to ensure accuracy. The device is small (25.5 x 28 x 9.65 mm), light (8 grams), has 4 to 6 months of battery life.

### Criterion Standard

Two researchers (BT, EB) observed patients during each session with a physical therapist. Similar to methods used in previous studies [17], the gold standard for the actual number of steps was the average of the two values counted by each researcher using a mobile counting app.

### Procedure With Accelerometer

#### Researchers

Patients were seen for standard inpatient physical therapy sessions with a licensed physical therapist and two researchers for the study. The two researchers were blinded to the type of surgery and the postoperative day.

#### Sensor Placement

**Figure 1:**
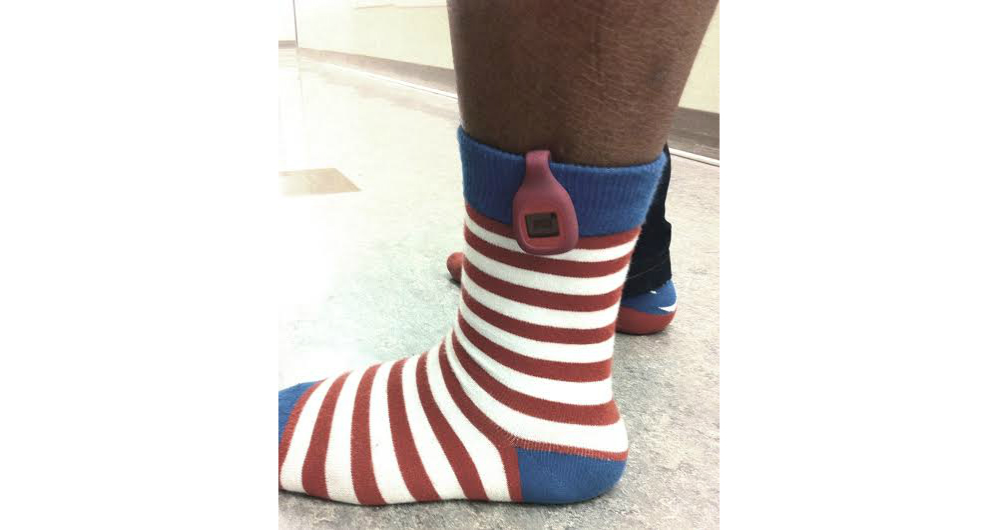
Placement of the activity sensor.

Four activity sensors were placed on each patient with one FitBit Zip at each of the following locations: on the right and left hips over the anterior superior iliac spine, as suggested by the manufacturer and by previous studies [22], and laterally over the right and left ankles (see Figure 1) due to hypothesized increased detection of movement. The sensors were placed on each patient immediately before beginning the course at the 0-meter starting line.

#### Walking Course

Each patient was asked to ambulate at a self-selected pace down a flat level course that was set up with the 0-meter, 4-meter, and 10-meter lines marked, and then further than 10 meters if deemed safe and appropriate by the physical therapist. If the patient walked further, this total distance was also recorded. Immediately after standing up from bed, the patients were asked to ambulate to the 0-meter starting line, which was always within 1 meter of the foot of their bed.

#### Data Collection

The gold standard number of steps was counted from the 0-meter to 4-meter line, 0-meter to 10-meter line, and the 0-meter line to the total distance if the patient ambulated further. A digital stopwatch with 1/10-second resolution was also used to record the time elapsed during the 0-meter to 4-meter and 0-meter to 10-meter intervals. The activity sensors recorded the number of steps taken for the total distance ambulated, and the reading from each of the four was documented. The 15-minute time interval corresponding to each physical therapy session was searched on the online account or the mobile app, and the number of FitBit-counted steps during that time was recorded; there were no overlapping intervals. Controls followed the same protocol except that they did not ambulate further than 20 meters.

### Clinical Variables

#### Sensor Accuracy

The primary outcome was the accuracy of the sensors in terms of mobility assessment, which was assessed by comparing the total number of steps recorded by each tracker to the total number of steps counted by the researchers. We also verified the time accuracy of the sensor by comparing the recorded times—available on both the Web interface and mobile phone app—to those recorded by the researchers.

#### Patient Demographics

Recorded information included postoperative day, type of procedure (ie, spinal surgery or craniotomy), postoperative diagnosis, presence and degree of weakness on standard neurological exam, age, and gender (see Table 1).

#### Level of Physical Assistance

To assess the level of physical assistance that the patient required to safely ambulate, the 6-point, graded Functional Ambulation Category (FAC) (see Multimedia Appendix 1) was used, which ranges from a score of 0 (patient is unable to independently ambulate) to 5 (fully independent) [23]. The FAC has been shown to predict ambulation ability in poststroke patients, and it correlates with other measures of functional recovery [24]. The FAC was determined in accordance with the same physical therapist at each session. It was also documented whether the patient used a rolling walker as an assistive device.

### Statistical Analysis

The total number of steps counted by the two researchers (gold standard) was compared to the steps recorded on the activity sensors using an intraclass correlation coefficient (ICC) [25]. This was performed for the sensors at all four bodily positions, the average of both ankles together, and the average of both hips together. The ICC was also calculated for subgroups based on the use of a rolling walker, FAC, and step length. To provide a more detailed analysis of the degree to which sensor accuracy is affected, the percent difference of sensor-recorded steps from the gold standard was calculated in terms of all four sensors, ankle average, and hip average. One-way analyses of variance (ANOVAs) were performed to compare the sensor difference from the gold standard, using the null hypothesis that the difference equals 0%. After an approximately normal distribution was verified, Student's *t* tests—one sample, paired sample, or independent samples where appropriate—were performed to compare the sensor differences from the gold standard, again using the null hypothesis that the difference equals 0%, between hips and ankles within the subject groups, and between subjects and controls. The chi-square test, ANOVA, Fisher's exact test, independent Student's *t* tests, and the Mann-Whitney U test were used when appropriate.

To identify independent predictors of accuracy, a multivariate model was conducted that included the following variables: age, gait speed, step length, postoperative day (POD), and surgical group. All statistical analyses were performed with SPSS version 21.

## Results

### Overview

There were a total of 148 motion data points generated from 37 individuals—27 patients and 10 controls—who met inclusion and exclusion criteria for enrollment in the study. Characteristics of the patient subjects are shown in Table 1. The 10 healthy controls had a median age of 27.5 years (interquartile range [IQR] 26.3-36.8), average gait velocity of 1.05 m/s (SD 0.83-1.19), and average step length of 0.646 m (SD 0.616-0.679). The devices recorded the correct date and time of all sessions, each lasting 10 to 15 minutes, with 100% accuracy. The data were visible on the mobile phones and were successfully uploaded online in 100% of cases.

**Table 1:** Characteristics of patient subjects (n=27).

Characteristic		Median (IQR^a^), n (%), or mean (SD)
Age in years, median (IQR)		57 (44-68)
Gender (male), n (%)		13 (48)
Walker used during session, n (%)		14 (52)
Average gait velocity (m/s)^b^, mean (SD)		0.260 (0.156-0.357)
Average step length (m)^b^, mean (SD)		0.232 (0.169-0.278)
Total distance walked (m), median (IQR)		50 (21-62)
Total steps ambulated^c^, median (IQR)		184 (127-255)
Postoperative day, median (IQR)		3 (2-5)
**Functional Ambulation Category (FAC)** ^d^ **, n (%)**		
	0	1 (4)
	1	4 (15)
	2	9 (33)
	3	13 (48)
**Surgical group, n (%)**		
	Spine	20 (74)
	Craniotomy	7 (26)
**Weakness (upper and/or lower extremity)** ^e^ **, n (%)**		
	Right-sided only	2 (7)
	Left-sided only	5 (19)
	Both	5 (19)

^a^Interquartile range (IQR).

^b^Calculated during the 4- or 10-meter walk.

^c^As determined by researchers using digital counting app.

^d^FAC is a measure of ambulation on a scale of 0 to 5; see Multimedia Appendix 1 for details.

^e^Determined by physician on standard neurological exam.

### Hip Versus Ankle Accuracy

In the subject group, the ankle sensors were more accurate in counting steps than the hip sensors when compared to the gold standard number of steps counted by the observers (ICC .837 vs .326, respectively). This inaccuracy was due to undercounting, since the hip sensors significantly underestimated the number of steps by -81.4% on average compared to the -26.1% underestimate seen in the ankle sensors (*P*<.001) (see Table 2). In approximately 50% of the subjects, one of the ankle trackers was accurate to within 15% (-15% to +15%). The underestimation in the subject group differed significantly from the respective ankle (*P*=.01) and hip (*P*<.001) recordings in the control group. Unlike in the subject group, the ankle and hip sensors in controls did not differ significantly from the gold standard, and they both had very good accuracy (ICC .890 and .863, respectively).

### Effect of Clinical Variables

**Figure 2:**
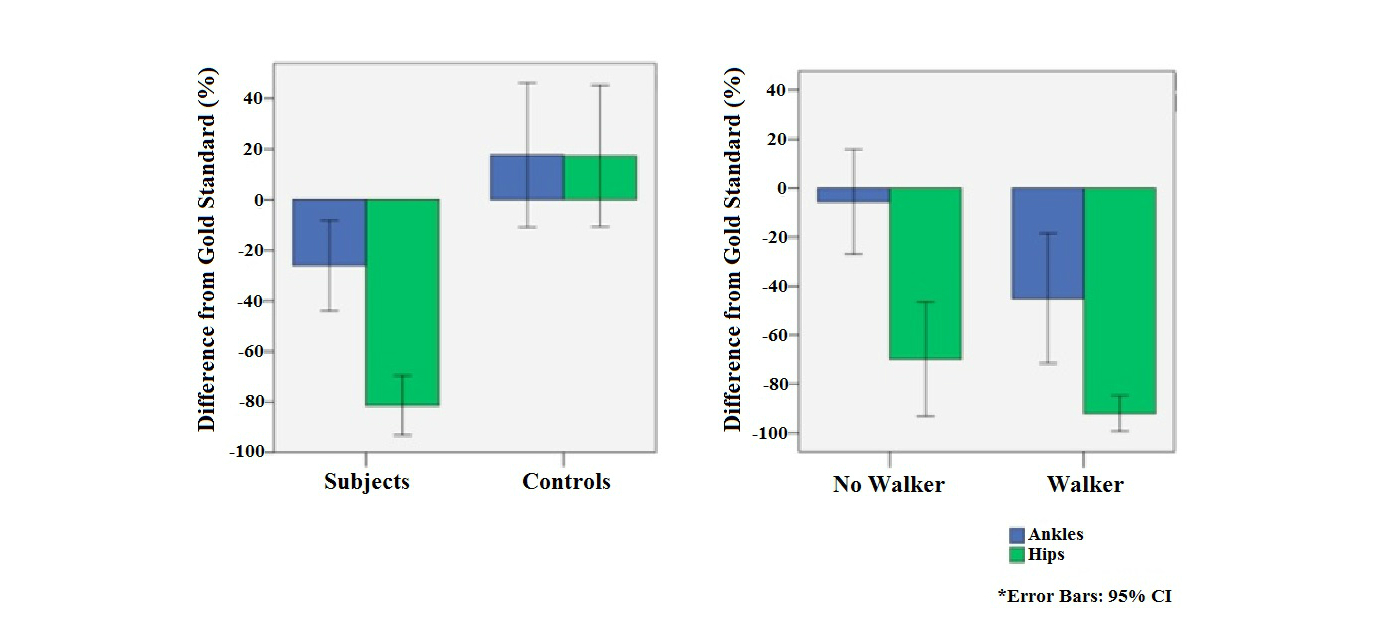
Mean differences in ankle and hip tracker recording in subjects versus controls (left); mean differences in ankle and hip tracker recordings in subjects with and without a rolling walker (right).

**Figure 3:**
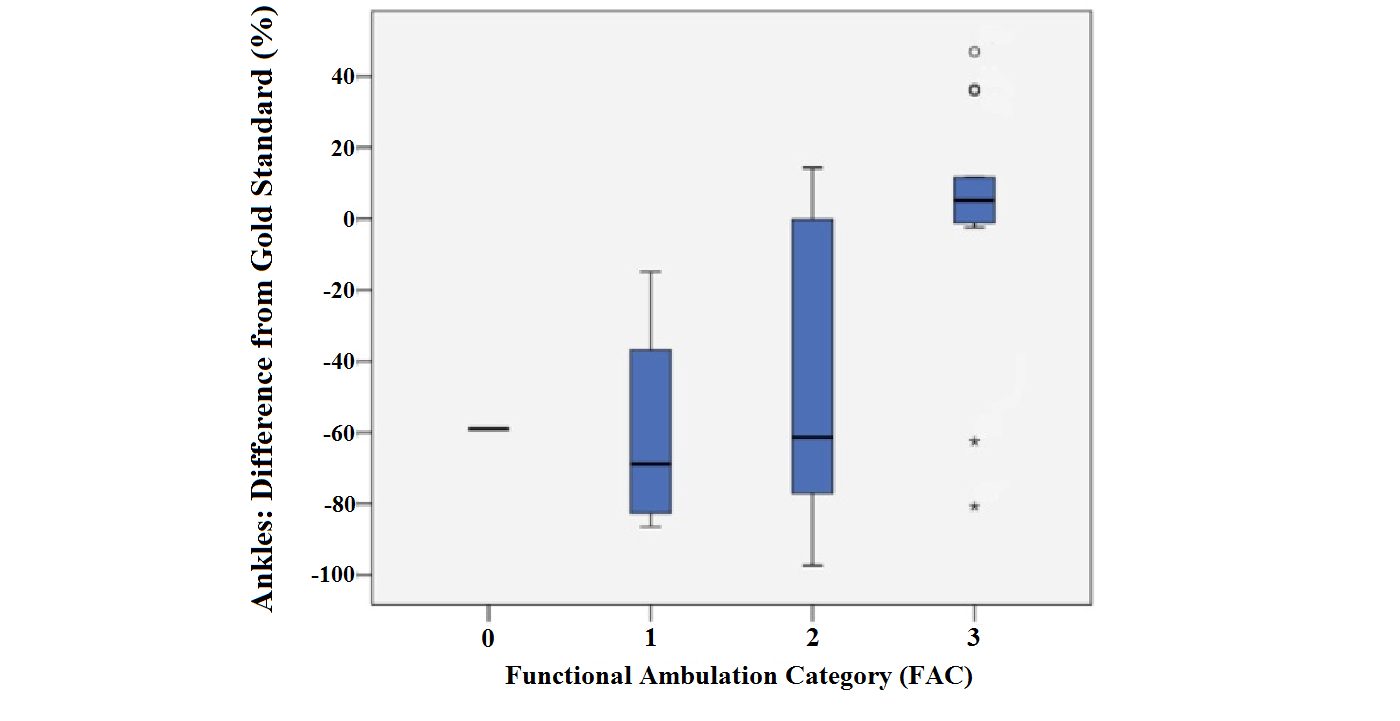
Functional Ambulation Category (FAC) in relation to ankle sensor mean differences from the gold standard.

Table 2 depicts the effect of the clinical gait variables on ankle tracker accuracy for the subject group. Although the ICCs appear comparable between the patients who used a rolling walker and those who did not, there was a significantly greater underestimation in the recordings for patients who used a walker than those without a walker (-45.1% vs -5.6%, respectively; *P*=.02) (see Figure 2). All subjects had an FAC ≤ 3, and the FAC appeared to affect the ankle tracker accuracy. Of the 27 subjects, 13 (48%) had an FAC of 3 (only standby guarding for potential falls), and 9 (33%) had an FAC of 2 (requiring assistance with balance or coordination). While the ICCs appear comparable for those subjects with an FAC of 3 or less, this was not the case for the mean difference. In the group with an FAC of 3, mean difference was only +0.78%, versus a significant underestimation in the more debilitated group with an FAC<3 which was -51.0% (*P*<.001). More specifically, as shown in Figure 3, there was a significant underestimation in the group with an FAC of 2 (-46.2%, 95% CI -80.3 to -12.1) compared to the group with an FAC of 3 (*P*=.02). Step lengths >0.232 m were more accurately tracked than step lengths that were shorter. An ICC of .973 was found for the longer step lengths compared to .792 in patients whose step lengths were shorter than 0.232 m. Compared to the gold standard, the smaller step lengths had a significant underestimation (-46.4%, 95% CI -70.4 to -22.4; *P*=.001), whereas for step lengths >0.232 m, the mean difference was not significantly different (+3.5%, 95% CI -13.5 to +20.6; *P*=.65).

**Table 2:** Intraclass correlation coefficient (ICC) and mean difference compared to the gold standard number of steps as counted by the researchers.

Sensor location and patient characteristic	ICC of number of steps (95% CI)	Mean difference, % (95% CI)	*P* (Student's *t* test or ANOVA^a^)
Hips—overall	.326 (-.214 to .684)	-81.4 (-93.2 to -69.5)	<.001 ^b^
Ankles—overall	.837 (.630 to .927)	-26.1 (-43.9 to -8.2)	.006
Ankles—without walker	.791 (.304 to .937)	-5.6 (-27.0 to +15.8)	.58
Ankles—with walker	.815 (.193 to .947)	-45.1 (-71.5 to -18.5)	.003
Ankles—with walker, with correction factor of +50%	.773 (.292 to .927)	-17.6 (-57.4 to +22.2)	.57
Ankles—step length >0.232 m	.973 (.902 to .993)	+3.5 (-13.5 to +20.6)	.65
Ankles—step length <0.232 m	.792 (.288 to .932)	-46.4 (-70.4 to -22.4)	.001
Ankles—step length <0.232 m, with correction factor of +50%	.734 (.238 to .907)	-19.6 (-55.5 to +16.3)	.29
Ankles—FAC^c^=3	.816 (.377 to .945)	+0.78 (-20.9 to +22.5)	.94
Ankles—FAC=0,1,2	.801 (-.080 to .949)	-51.0 (-73.3 to -28.7)	<.001
Ankles—FAC=0,1,2, with correction factor of +50%	.803 (.387 to .937)	-26.5 (-59.9 to +7.02)	.15

^a^Analysis of variance (ANOVA).

^b^Values in italics are statistically significant.

^c^Functional Ambulation Category (FAC).

### Correction Factor

To show that the undercounting bias could be adjusted in patients with an FAC< 3, step length <0.232 m, and those using a walker, we added 50% to the original step counts in these subgroups—the approximate underestimation in each case—and mean difference from the gold standard improved significantly (Table 2).

### Multivariate Analysis

**Figure 4:**
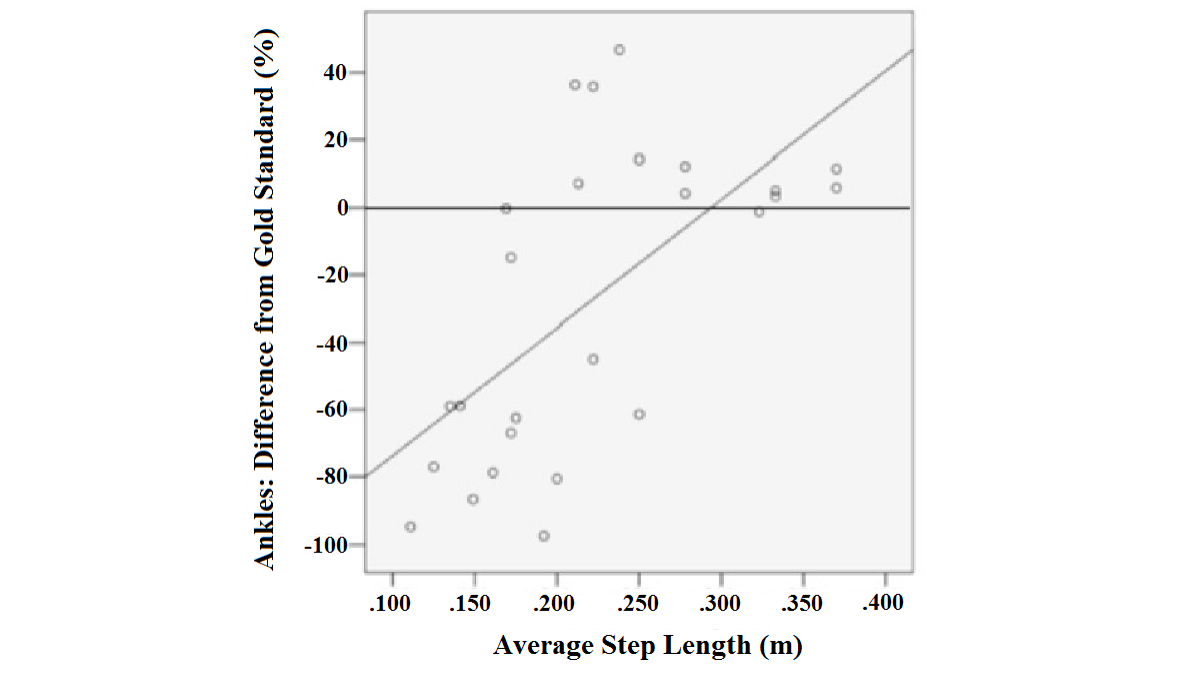
Scatterplot of ankle sensor differences in relation to average step length.

Table 3 demonstrates the multivariate analysis of clinical variables that found step length to be an independent predictor of overall tracker accuracy (*P*=.03). Figure 4 shows that there was a modest positive correlation between longer step length and improved ankle tracker accuracy (*r*=.640, *r* ^2^=.397). Although increased gait speed also correlated with increased ankle sensor accuracy, the relationship was weaker (*r*=.444, *r* ^2^=.197), and it lost statistical significance when we controlled for step length in the multivariate analysis.

**Table 3:** Multivariate analysis of predictors of ankle sensor accuracy.

Variable	*P*
Age	.81
Postoperative day (POD)	.55
Gait speed	.44
Step length	.03^a^
Surgical group	.75

^a^Values in italics are statistically significant.

There were no significant differences observed between right-sided and left-sided trackers when comparing subjects with left-sided versus right-sided weakness. The total distance ambulated also did not significantly affect the accuracy of the ankle trackers (*P*=.39).

## Discussion

### Principal Findings

We assessed the practicality and reliability of wearable, easy-to-use activity sensors in patients with limited mobility in the early postoperative period. Data from the rehabilitation sessions were remotely accessible by an online or mobile phone interface—an unprecedented technology that will provide health care professionals with the amount, duration, and timing of patient mobility at home and in the hospital. Although the activity sensors accurately tracked the time and duration of each session, in terms of step counting, our results highlight that ankle versus hip sensor placement, along with specific characteristics of patient mobility—use of an assistive device, step length, and FAC—affect the devices’ ability to accurately reflect patient functional recovery.

### Gait Parameters and Comparison to Previous Studies

As mentioned earlier, mobilization generally improves survival and functional outcome in a wide variety of patients recovering from surgery, neurological illness, and cancer, but accurately tracking mobility, especially in the outpatient setting, has been challenging. A patient’s mobility can be graded by physical performance measures such as gait speed, which is a function of age, stature, and strength [26]. Patients who are more disabled tend to have slower gait speeds and have a higher risk of hospitalization and death related to immobility [2,8,12]. In a large longitudinal study of 34,000 adults, each 0.1 m/s increase in gait speed was independently associated with a lower risk of death with a hazard ratio of 0.90 (95% CI 0.89-0.91; *P*<.001) [12]. Although our subjects were relatively functional compared to many neurology and neurosurgery patients, who are often very debilitated in the acute period, our study is among the first to assess the use of commercial, wearable sensors in patients with very slow gait speeds (IQR 0.156-0.357 m/s) and limited mobility. Most other studies have had a lower limit of 0.500 to 0.580 m/s [2,9,19] and, therefore, have not provided sufficient data on patients at risk of harmful consequences associated with decreased gait speed.

### Sensor Reliability in Relation to Functional Status

We observed an underestimation of step counts in the less mobile subject population, likely because these patients tended to have a lower FAC, shorter step length, and need for a walker. These common clinical characteristics made patients’ movements less pronounced, which were more difficult for the sensors to detect—especially those placed on the hips. On the other hand, the readings in the control group did not significantly differ from the gold standard, indicating that the sensor accuracy was greater than in the subject group. The higher accuracy in the control group, who had gait speeds in the normal range [26], was probably because those individuals had larger, more pronounced movements during ambulation which were more easily detected by the sensors. As a result, the placement of sensors on the hips (as suggested by the company) resulted in more accurate readings in controls because of normal, detectable hip motion that may be less pronounced in recovering patients. These results were not unanticipated, since the devices are marketed toward healthy, active individuals. That being said, we did note a degree of overestimation in the control group that was not statistically significant.

The tendency to underestimate in more slowly moving patients was also observed in a study where sensors undercounted by 19.1% to 32.1% when gait speeds were ≤0.800 m/s [27]. In one of the few studies that included patients with slow gait speeds, it was observed that a specialized, noncommercial sensor was more accurate in the control group but undercounted in older, frail stroke patients with gait speeds ≤0.470 m/s [28]. In our study, although accuracy was related to gait speed, there was a stronger relationship to step length. In patients with a step length >0.232 m, the sensors were highly accurate compared to the gold standard (ICC .973, 95% CI .902-.993). Step length is known to have an effect on clinical characteristics, as it decreases with age and certain orthopedic injuries, and can influence postural stability [29]. As a clinical measure, it remains understudied compared to gait speed, although the two are physically and inherently related. Step length, therefore, may provide another important measure of a patient’s mobility.

Within the subject group, sensor accuracy was strongest in patients who were more mobile, including those who ambulated without a walker or significant assistance from the physical therapist and, as mentioned, those with longer step lengths. This inverse relationship between amount of movement and degree of undercounting suggests that the sensors underestimate more as a patient’s mobility and functional status worsen. The relationship between decreased accuracy of other accelerometers and poorer functional status, such as in patients with congestive heart failure and chronic obstructive pulmonary disease (COPD), was noted in a systematic review on the validity of activity sensors in patients with chronic disease [21]. Decreased mobility, in turn, is associated with poorer outcomes as described earlier. For this reason, a reliable assessment of mobility in this patient population is needed in order to monitor recovery and detect a decline in health that would require intervention.

### Limitations

Some limitations of this study should be mentioned. Since this was a pilot study, our sample size was 27 subjects, although this is in the range of comparable previous studies [17-19,27,29,30], and we found a large effect size. Our data also had higher resolution than would be expected from the sample size because it included 148 readings from the sensors. As with previous studies, there was the possibility of human error in step counting, although this remains the gold standard. It may be stated that our results are less generalizable because all subjects were neurosurgical patients with postoperative gait impairment, but patients with preexisting gait disorders were excluded, and we found no effect of unilateral weakness on sensor accuracy. Since there is variation in the technology and software used to detect physical activity, measurements among sensors are not necessarily consistent with each other, and for this reason we only used one type of device. Regarding the device which was used in the study, FitBit Zip, it should also be mentioned that it has not yet been approved for medical purposes. It is only a matter of time, however, until wearable, commercially available activity sensors are used to improve patient care, as these sensors are far more affordable than comparable devices and have the potential for widespread use [21].

### Conclusions

In conclusion, activity sensors generate low-cost data on patient recovery in the hospital and in the home environment. We have shown that they are able to remotely monitor patient activity, although our results demonstrate that in order to develop research protocols which use activity sensors to reliably track patient mobility, the suitability of the sensors needs to be individually determined, as suggested by previous authors [31]. The technical characteristics of the accelerometer must be considered, and care must be taken when interpreting the results of data recorded on the devices. Additionally, in patients with limited mobility, one must clinically and mathematically account for certain factors—FAC, step length, use of assistive device—in order to use wearable sensors as a means of accurately assessing the degree of disability. The positioning of the sensor should also be carefully considered depending on the degree of to-and-fro movements. Our data may be utilized to determine the best way to standardize the use of activity sensors, and eventually provide the missing outpatient data needed to assess functional recovery.

### Future Directions

Since impaired ambulation and limited mobility may occur in a wide variety of other diseases [12]—cardiovascular, pulmonary, musculoskeletal—wearable sensors have extensive potential across a broad range of medical fields. With the determinants of accuracy that we have outlined, sensors may soon be used not only by health care professionals to supplement acute care, but also by patients at home after discharge. Patients can wirelessly track their own recovery and mobility using feedback provided on the display screen of certain wearable devices, which provide real-time snapshots of activity level—number of steps taken, calories burned, and distance covered. This would allow patients to engage in their own recovery by aiming to achieve certain activity goals [32,33], and they may be further motivated to do so with the knowledge that they are being remotely monitored by their health care provider. In this manner, the activity sensors may prove to be a form of therapeutic intervention which promotes improvement in mobility and independent function [34,35]. In effect, reliable use of activity sensors will not only add to the repertoire of inpatient physical therapy measures, but will also provide much-needed longitudinal data to track patients as they recover in the outpatient setting. It will be possible for health care professionals to log on to a Web portal and see not only the trends of a patient’s mobility over the course of weeks to months, but also the minute-to-hour variations in activity throughout the day from which bed rest or inactivity could be inferred. In fact, a similar accelerometer has been used to track patients for several days after having received cardiac surgery [36]. Using information from our research, future studies will be able to develop a reliable protocol for using wearable sensors and enroll patients on a large scale. Outpatient data from the sensors could then be compared to a variety of standard outcome measures such as the Modified Rankin Scale, Barthel Index, and quality-of-life scales.

## Multimedia Appendix 1

Functional Ambulation Category (FAC).

## Abbreviations

ADL: activities of daily living
ANOVA: analysis of variance
COPD: chronic obstructive pulmonary disease
FAC: Functional Ambulation Category
HIPAA: Health Insurance Portability and Accountability Act
ICC: intraclass correlation coefficient
ICU: intensive care unit
IQR: interquartile range
IRB: Institutional Review Board
POD: postoperative day
