# Supplementary material for: Mobile Phone-Connected Wearable Motion Sensors to Assess Postoperative Mobilization
Source: JMIR Mhealth Uhealth. 2015 Jul 28;3(3):e78. doi: 10.2196/mhealth.3785 (PMC4705357; doi:10.2196/mhealth.3785)
Supplement: Supplementary file 1 [file mhealth_v3i3e78_app1.pdf]

Appendix—Description of Functional Ambulation Category (FAC)

FAC	Ambulation Description	Definition
0	Nonfunctional ambulation	Subject cannot ambulate, ambulates in parallel bars only, or requires supervision or physical assistance from more than one person to ambulate safely outside of parallel bars
1	Ambulator-Dependent for Physical Assistance Level II	Subject requires manual contacts of no more than one person during ambulation on level surfaces to prevent falling. Manual contacts are continuous and necessary to support body weight as well as maintain balance and/or assist coordination
2	Ambulator-Dependent for Physical Assistance Level I	Subject requires manual contact of no more than one person during ambulation on level surfaces to prevent falling. Manual contact consists of continuous or intermittent light touch to assist balance or coordination
3	Ambulator-Dependent for Supervision	Subject can physically ambulate on level surfaces without manual contact of another person but for safety requires standby guarding on no more than one person because of poor judgment, questionable cardiac status, or the need for verbal cuing to complete the task.
4	Ambulator-Independent Level Surfaces only	Subject can ambulate independently on level surfaces but requires supervision or physical assistance to negotiate any of the following: stairs, inclines, or non-level surfaces.
5	Ambulator-Independent	Subject can ambulate independently on nonlevel and level surfaces, stairs, and inclines.
